# Hypothesis for a mechanism of beam-induced motion in cryo-electron microscopy

**DOI:** 10.1107/S2052252520002560

**Published:** 2020-03-26

**Authors:** Robert E. Thorne

**Affiliations:** a Physics Department, Cornell University, Ithaca, NY 14853, USA

**Keywords:** cryo-EM, beam-induced motion, radiation damage

## Abstract

Estimates of heat-transfer rates during plunge-cooling and the patterns of ice observed in cryo-EM samples indicate that the grid bars cool much more slowly than do the support foil and sample near the middle of the grid openings. The resulting transient temperature differences generate transient tensile stresses in the support foil. Most of this foil stress develops while the sample is liquid and cooling toward its glass transition *T*_g_, and so does not generate tensile sample stress. As the grid bars continue cooling towards the cryogen temperature and contracting, the tensile stress in the foil is released, placing the sample in compressive stress. Radiation-induced creep in the presence of this compressive stress should generate a doming of the sample in the foil openings, as is observed experimentally. Crude estimates of the magnitude of the doming that may be generated by this mechanism are consistent with observation. Several approaches to reducing beam-induced motion are discussed.

## Abstract 2

Transient temperature differences between the grid and support foil during cooling may generate compressive stresses in the sample that drive the observed beam-induced doming motion.

## 1. Introduction   

In biomolecular single-particle cryo-electron microscopy, biomolecules in aqueous solution are deposited and are then blotted or wicked to form a thin film on a thin holey carbon or metal support foil, which is in turn supported by a much thicker metal grid (Cheng *et al.*, 2015 ▸). These samples are rapidly cooled by plunging in liquid ethane, so as to vitrify the biomolecule-containing film. Upon irradiation by electrons, the biomolecules (and the amorphous ice in which they reside) within the holes in the foil are observed to move (Glaeser, 2016 ▸; Vinothkumar & Henderson, 2016 ▸). This motion causes blurring of the acquired images (Glaeser *et al.*, 2011 ▸; Brilot *et al.*, 2012 ▸; Russo & Passmore, 2016*a* ▸,*b* ▸; Glaeser, 2016 ▸; Henderson, 2018 ▸; Shi *et al.*, 2019 ▸). High-efficiency, high-frame-rate direct electron detectors have allowed movies of this motion to be acquired (Campbell *et al.*, 2012 ▸; Faruqi & McMullan, 2018 ▸), and algorithms have been developed to model and correct for the effects of this motion (Li *et al.*, 2013 ▸; Rubinstein & Brubaker, 2015 ▸; Ripstein & Rubinstein, 2016 ▸; Zheng *et al.*, 2017 ▸). These advances have contributed to dramatic improvements in the achievable resolution in cryo-EM and to an explosion of interest in this approach to biomolecular structure determination.

However, beam-induced sample motion remains a serious factor limiting the achievable resolution and the interpretation of acquired images. Experimental studies to date suggest the following salient features. Firstly, motion is a function of dose, not time. Secondly, the rate of change of particle positions with dose is largest at low doses, below 2–4 e^−^ Å^−2^ (Vinothkumar & Henderson, 2016 ▸), when accumulated radiation damage is modest and the highest-resolution structural information is available. The direction and magnitude of motion varies with position in each hole and between holes, but the largest motion component corresponds to a doming of the vitrified sample within the hole (Wright *et al.*, 2006 ▸; Brilot *et al.*, 2012 ▸; Campbell *et al.*, 2012 ▸; Zheng *et al.*, 2017 ▸). The amount of motion appears to be reduced by reducing the size of the holes in the foil (Russo & Passmore, 2016*b* ▸).

Electron irradiation breaks bonds and increases the average distance between atoms, so that the sample film is expected to expand with dose (Vinothkumar & Henderson, 2016 ▸). The expansion should be roughly linear with dose (except at large doses, where damage becomes severe), as is found in X-ray crystallography (Ravelli *et al.*, 2002 ▸). This mechanism cannot explain the rapid initial motion, but may account in part for the more gradual motion at larger doses.

Electron irradiation may promote the release via plastic creep of stress that is developed within the sample during cooling. Creep is a gradual plastic deformation that occurs when a sample is subjected to stresses well below its yield stress. Irradiation-induced creep has been extensively studied, for example in the context of materials properties for nuclear reactors (Bullough & Wood, 1980 ▸; Shibata, 2013 ▸). Creep is largest at the start of irradiation, when the stress is largest, and decreases as irradiation continues and the driving stress is relaxed by creep. This mechanism provides a natural explanation for initial rapid beam-induced motion in single-particle cryo-EM samples. However, the nature and origin of the driving stress has not been identified.

Here, we discuss a possible origin for cooling-induced stress in cryo-EM samples that is qualitatively consistent with observation, and how this stress may be reduced.

## 2. Mechanisms for sample stress generation   

### 2.1. Cooling a biomolecule-containing sample on a freely sliding support foil generates tensile sample stress   

Suppose that a biomolecule-containing sample is deposited on a holey foil, and that the foil is somehow able to freely slip relative to the supporting grid (supporting information, Section S1). In this case, the contraction of the foil during cooling is not coupled with that of the grid. Consequently, as the foil cools, it will contract at a temperature-dependent rate that depends only on its composition and its interaction with the sample.

The behavior of the biomolecule-containing sample on cooling is more complex. Between room temperature and the glass-transition temperature of water, *T*_g,water_ ≃ 136 K (Amann-Winkel *et al.*, 2016 ▸), water undergoes a net volume expansion of ∼8%. Since the sample is liquid in this temperature range, this expansion is essentially uncoupled from the contraction of the support foil. Consequently, differences in the thermal contraction of the sample and foil between room temperature and *T*_g_ should generate no sample stress^1^.

On cooling from *T*_g_ to the final temperature *T*_cryo_ (typically ∼90 K), the thermal contraction of bulk amorphous ice roughly matches that of hexagonal ice I_h_, which has an average linear thermal expansion coefficient over this temperature range of 1.5 × 10^−5^ K^−1^ (Röttger *et al.*, 1994 ▸; supporting information, Section S2). Average thermal expansion coefficients over the same temperature range are smaller for all metals and other materials (amorphous carbon, silicon and silicon nitride) used as sample supports in cryo-EM; the average expansion coefficients of gold (∼1.2 × 10^−5^) and copper (∼1.1 × 10^−5^) (Corruccini & Gniewek, 1961 ▸) provide the closest match to that of I_h_ in this temperature range. Thus, the differences in thermal expansion coefficients between the sample and a freely sliding support foil should result in tensile sample stress once both have cooled to *T*_cryo_. Beam-induced sample creep would then produce thinning, rather than the observed doming, of the sample within the holes in the foil.

### 2.2. The sample-support foil and grid are tightly coupled   

In fact, the foil is in general not free to slip on the grid, but is tightly fixed to it by dispersion and electrostatic forces, by surface-tension forces from sample that flows through the holes in the foil or from the front to back of the foil during sample deposition and blotting and wets the grid bars, by surface-tension forces from ambient moisture that may condense on the grid and foil, and (once sufficiently cold) by vitrified or crystalline ice.

### 2.3. When the foil is in tension, its dimensions must match those of the grid   

If the grid is stretched, the foil must stretch with it (at least until the tension of the foil reaches the limit of static friction between the foil and the grid). If the grid is compressed, the foil will buckle where it is not in contact with the grid. The grid is much thicker than the foil (∼10 µm versus ∼0.05 µm), and so has much greater elastic stiffness. Consequently, the thermal contraction of the grid material alone will (to a first approximation) determine the dimensions of the foil at all temperatures, as long as the foil is in tension. Tension and buckling of the foil at the final temperature *T*_cryo_ can be minimized by using the same material (for example gold) for the grid and foil (Russo & Passmore, 2016*a* ▸).

### 2.4. If the grid, foil and sample had the same temperature during cooling, differences in the thermal expansion coefficients of the grid, foil and sample would determine the sample stress   

If the grid material has a larger average thermal expansion coefficient between *T*_cryo_ and 300 K than the foil, on cooling to *T*_cryo_ the foil (and sample) will buckle. If the grid has a smaller average thermal expansion coefficient, the physical contraction of the foil will match that of the grid, and the foil will develop tension. The stress developed in the sample will then be determined by the mismatch between its average thermal expansion coefficient between *T*_g_ and *T*_cryo_ and that of the grid (not the foil). Pure amorphous ice has a larger average expansion coefficient in this temperature range than all materials used in cryo-EM grids (supporting information, Section S3). Consequently, if the grid, foil and sample all had the same temperature during cooling, the sample would always be under tensile stress, and no doming upon irradiation would be expected.

### 2.5. During plunge-cooling, grids cool more slowly than the support foil and biomolecule-containing sample   

Pure water vitrifies when cooled at rates in excess of ∼250 000 K s^−1^ (Warkentin *et al.*, 2013 ▸). Routine observation of crystalline ice on and near the grid bars even when the sample on the foil distant from the grid bars is fully vitrified provides direct evidence that grid bars cool more slowly.

In the boundary-layer approximation for forced convective heat transfer, the coefficient of heat transfer *h* from the grid, foil and sample to the liquid cryogen depends only on the plunge speed, the grid dimensions and the properties of the liquid cryogen (Kriminski *et al.*, 2002 ▸). The rate of heat transfer per unit area of foil and grid depends only on *h* and on the difference between the local grid/foil/sample temperature *T* and *T*_cryo_, d*q*/d*t* = *h*(*T* − *T*_cryo_). If the grid and foil were thermally isolated from each other, the cooling rates of each would then be proportional to their heat capacity per unit area. For a 50 nm gold foil on a 10 µm thick gold grid, these differ by a factor of ∼10^3^ (supporting information, Section S4).

Thermal conduction through the foil and sample from the grid bars to the center of the foil in each grid opening (as well as heat transfer from the grid to the liquid cryogen that then flows over the adjacent foil) will reduce the cooling rate of the foil and sample towards that of the grid. With nonmetal (for example amorphous carbon) foils, the thermal conductance κ of the foil and sample is small (supporting information, Section S5), and the rate of heat transfer from grid bars through the foil per unit area [d*q*/d*t* ≃ (κ*t*/*w* ^2^)(*T*_grid_ − *T*_foil_), where *w* is the width of a grid square and *t* is the foil thickness] will always be small compared with the rate of heat transfer from the sample and foil to the liquid cryogen. With (high thermal conductivity) gold foils, the rates of heat transfer per unit temperature difference from grid to foil and from foil to liquid cryogen are more nearly comparable, *i.e.* *h* ≃ κ*t*/*w* ^2^. However, this still implies that large temperature differences between the grid bars and the sample and foil near the center of each grid opening (and large temperature gradients within the sample and foil) must transiently occur during cooling, and that the grid bars will reach the final temperature *T*_cryo_ long after the sample and foil near the center of each grid opening.

The thermal mass near the grid bars is further increased by the common presence of excess unwicked sample that flows through foil holes and wets the back side of the foil and the grid bars. Between room temperature and 90 K, the total heat capacity per unit *volume* of water/hexagonal ice is roughly 50% more than that of gold (supporting information, Section S5), and so excess sample on the grid bars can substantially slow their cooling. Ice is a poor thermal conductor, with a conductivity ∼10^−2^ times that of gold, and so has only a modest effect on thermal conduction from grid bars to the foil center.

### 2.6. Slower cooling of the grid produces transient tensile stress in the support foil   

**Figure 1:**
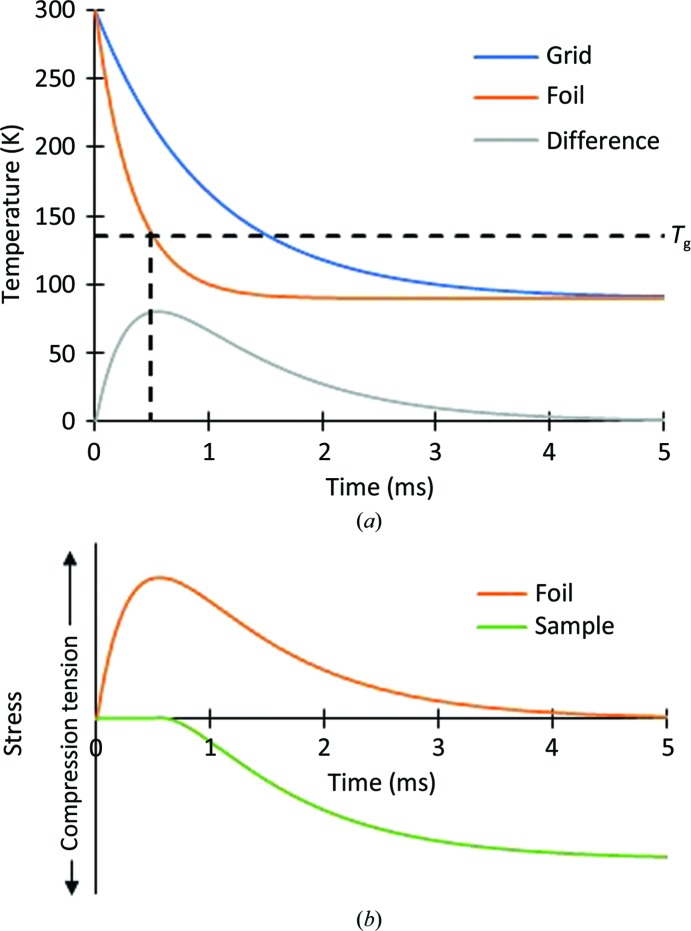
Temperature and stress during plunge-cooling of cryo-EM samples. (*a*) The grid cools more slowly than the foil and sample near the middle of the grid openings, so a large temperature difference between the grid and foil may transiently occur. (*b*) The transient temperature difference produces a transient tensile stress in the foil, the dimensions of which are constrained by those of the grid. The sample vitrifies and becomes strongly coupled to the foil only at *T*_g_, when the foil is under tensile stress. As the grid bars cool, the temperature difference between the grid bars and foil decreases and the tensile stress in the foil is released, the sample is placed under compressive stress. The grid and foil are here assumed to be of the same material.

As shown in Figs. 1 ▸ and 2 ▸, slower cooling of the grid bars than the foil and sample between them must produce transient tensile stress in the foil. The foil tries to contract toward the equilibrium length appropriate to its temperature, but is prevented from doing so by the more rigid grid that has cooled and contracted less. This is true even if the grid and foil are of the same material. The biomolecule-containing sample then vitrifies on a foil that is already under tensile stress. If the cooling rate of the grid bars is much smaller than that of the sample and foil between them, the temperature difference between the grid bars and foil may continue to grow, the tensile stress in the foil may grow, and the sample may also develop a small amount of tensile stress as it cools from *T*_g_ to *T*_cryo_.

### 2.7. Transient tensile stress in the foil is released as the temperature of the grid and foil converge to the final *T*_cryo_   

As the grid continues to cool towards the final temperature *T*_cryo_ of the grid and foil, the temperature difference and the difference in equilibrium length of the grid and foil eventually decrease, and the tensile stress in the foil decreases. If the grid and foil are of the same material, the stress in the foil (in the absence of sample) will be completely released once they both reach *T*_cryo_.

### 2.8. Release of transient tensile foil stress places the sample under compressive stress   

The sample vitrifies on a foil that is under tensile stress because the foil is colder than the grid bars. As the temperature difference between the grid bars and foil goes to zero, the contracting grid allows the foil to contract toward its equilibrium length, and the foil stress is released. As the foil attempts to contract, this places the vitrified sample upon it under compressive stress.

### 2.9. Radiation-induced sample creep in the presence of this compressive stress may generate doming of the sample within the holes in the foil   

When stressed (loaded), irradiated materials undergo creep in a way that releases the stress (Bullough & Wood, 1980 ▸; Shibata, 2013 ▸). In a simple model, the irradiation-induced creep strain ∊_c_ is given by ∊_c_ = *a*σ[1 − exp(−*bD*)] + *c*σ*D*, where *D* is the radiation dose, σ is the stress and *a*, *b* and *c* are constants. In the presence of compressive sample stress parallel to the plane of the foil, the sample may release that stress by increasing its area via a doming motion, as occurs when, for example, a ruler is squeezed at its ends along its length.

For samples within foil holes of diameter 1.2 µm, Brilot *et al.* (2012 ▸) observed radiation-induced doming displacement of the sample, with a displacement of the central region perpendicular to the plane of the foil of ∼150 Å after a dose of 32 e^−^ Å^−2^. The ratio of the surface area of a spherical cap of radius *a* and height *h* to the area of a disk of the same radius is 1 + *h* ^2^/*a* ^2^, and so the observed doming corresponds to an ∼0.06% increase in sample area. Crudely, we can assume that this increase in area is comparable to the fractional decrease in hole area from when the sample vitrifies to when the grid reaches the same final temperature *T*_cryo_ as the sample and foil. If the grid and foil are both made of gold, which has an average areal expansion coefficient of ∼2.5 × 10^−5^ between room temperature and 90 K (Corruccini & Gniewek, 1961 ▸), then this change in hole area would be associated with a modest 25 K temperature difference between the grid and the sample and foil (near the grid-opening centers) at the time of sample vitrification. The actual temperature differences could be as large as ∼160 K, and this analysis neglects any contribution to doming within the holes from radiation-induced creep of compressively stressed sample outside the holes. Consequently, both the sign and the magnitude of the observed sample motion appear to be consistent with the present model.

## 3. Reducing beam-induced motion   

Based on the mechanism for rapid initial beam-induced motion discussed here, how might this motion be reduced?

Firstly, the support foil and grid can be designed so that the foil can slip relative to the grid (at least in some regions of the grid), releasing tensile stresses that arise owing to slower cooling of the grid than the foil. The limit of static friction between the foil and grid will depend on the choice of the foil and grid materials, their surface roughnesses and their surface cleanliness/oxidation state. Inert layered materials such as graphite or MoSe_2_ might yield suitably low friction. A support foil material with a larger elastic modulus than gold or holey/amorphous carbon could promote foil slip over deformation. Using a continuous rather than holey foil or a foil covered with a thin graphene film may also help by preventing wetting of the sample to the grid bars and gluing of the grid and foil by vitrified or crystallized sample. Reduced sample motion using graphene-covered sample supports has recently been reported (Naydenova *et al.*, 2019 ▸).

Secondly, as has already been demonstrated (Russo & Passmore, 2016*a* ▸), the hole size in the supporting foil can be reduced. Beam-induced sample creep in the presence of stress is likely to be constrained by contact with the supporting foil, so creep (and certainly doming) may be largely confined to the holes. In the simplest model, the release of a given amount of stress Δσ (an intensive quantity) will produce a fractional change in linear dimension of Δ*L*/*L* and in area of Δ*A*/*A* ∝ (Δ*L*/*L*)^2^. When a flat disk of radius *a* expands to a spherical cap of height *h* and the same base radius, its surface area changes by Δ*A*/*A* = (*h*/*a*)^2^. Consequently, *h*/*a* ∝ Δσ, and the dome height will decrease with decreasing hole size.

Thirdly, the grid can be made of a material that undergoes only modest thermal contraction on cooling. Molybdenum, tungsten and doped silicon have thermal expansion coefficients that are roughly 1/3, 1/3 and 1/15, respectively, of those of copper and gold. This will reduce the maximum foil stress owing to transient temperature differences between the foil and grid, and thus the compressive sample stress generated when their temperatures converge.

Fourthly, temperature differences between the foil and grid during cooling can be reduced by reducing the difference in their cooling rates. This can be accomplished by reducing the heat capacity of the grid, by using a grid material with a lower specific heat per unit volume, or by reducing the width and/or thickness of the grid bars. Regions of reduced grid thickness/width, where imaging will be performed, can be mechanically and thermally decoupled via ‘weak links’ from surrounding regions needed to provide mechanical stiffness for handling.

Finally, the temperature to which the sample is initially cooled can be increased by raising the temperature of the liquid ethane. As the foil cools more rapidly and reaches the final temperature much sooner than the grid, this will decrease the maximum temperature difference between the foil and the grid, and reduce the release of tensile stress in the foil as the grid cools to the final temperature.

The glass transition of pure water is at 136 K, and it undergoes devitrification on warming above ∼150 K (Mayer & Hallbrucker, 1987 ▸; Hallbrucker & Mayer, 1987 ▸; Jenniskens & Blake, 1996 ▸). To ensure that the cooling time to below *T*_g_ is sufficiently short to achieve vitrification, the final temperature must be well below *T*_g_; how much below will depend on the sample and foil thicknesses, and also on the maximum ice fraction in the sample that is tolerable to achieve high-resolution imaging.

For aqueous solutions, the glass-transition temperature increases and the critical cooling rate required to achieve vitrification decreases with increasing solute concentration (Warkentin *et al.*, 2013 ▸). Consequently, increasing the solute concentration should allow higher ethane temperatures to be used. The solutes can include salts present in protein buffers as well as cryoprotectants such as glycerol. However, these reduce the electron-density contrast between the biomolecule and buffer (Tyree *et al.*, 2018 ▸), and so reduce the measurement signal to noise. Solutes also include the biomolecules themselves. These tend to be less effective in suppressing ice formation on a per-unit-mass basis than salts or glycerol. However, if they can be highly concentrated without aggregation, including via evaporation after deposition on the grid, they could be quite effective.

Very recently, Shi *et al.* (2019 ▸) reported improved cryo-EM image resolution for apoferritin at low doses, suggesting reduced beam-induced motion in the early stages of the exposure, using samples cooled in ethane at 163 K. These results are consistent with the mechanism for beam-induced motion discussed here. The very high temperatures used, which are well above *T*_g_ for water and dilute aqueous solutions, are highly unlikely to yield ice-free samples under most conditions. Shi and coworkers only report fast Fourier transforms of real-space images, which provide much less sensitive detection of crystalline ice than direct diffraction measurements, so the presence of ice in their samples cannot be ruled out. However, the particle densities in their images are very high, and may have been sufficient to allow vitrification at such high temperatures.

## 4. Vitrification at elevated temperatures?   

Shi and coworkers reported protein concentrations in the deposited liquid of ∼2 mg ml^−1^ or ∼0.2%(*w*/*w*). This is far too small to appreciably modify the glass-transition temperature of the solution (Angell, 2002 ▸) or the critical cooling rates for vitrification (Warkentin *et al.*, 2013 ▸), and the fraction of solvent within the first two hydration layers of the protein molecules (which is unlikely to crystallize; Moreau *et al.*, 2019 ▸) at this concentration is also small. However, the cryo-EM images reported by Shi and coworkers (and those often obtained in general cryo-EM practice) suggest that the final protein concentrations after blotting and evaporation and that are captured in the plunge-cooled samples are much larger than in the original solution.

For a solution with a protein concentration *c* in mg ml^−1^, the number of biomolecules per nm^3^ is 0.6*c*/MW, where the molecular weight MW is in g mol^−1^. For a sample film of thickness *t* in nanometres, the number of biomolecules per nm^2^ is 0.6*ct*/MW. Assuming an average protein density of 1.35 g ml^−1^, the projected area in nm^2^ of each molecule is ∼0.014(MW)^2/3^. The total fraction of the area occupied by molecules in a 2D projection through a film of thickness *t*, neglecting any overlap of their projections (valid at low concentrations), is then 0.0083*ct*(MW)^−1/3^. For apoferritin (MW = 480 kDa) at *c* = 2 mg ml^−1^ in a film *t* = 50 nm thick, the fraction of the area occupied by molecules should then be ∼1%. In Fig. S2 of Shi *et al.* (2019 ▸), roughly 65% of the area is occupied by apoferritin molecules. This corresponds to a concentration *c* of ∼130 mg ml^−1^, or roughly 13%(*w*/*w*). Any salts that are present in the initial buffer may be concentrated by a similar factor, as they tend to associate with the bio­molecule. This concentration estimate assumes a 50 nm sample thickness, but based on the hexagonal ordering of the apoferritin particles in Fig. S2 of Shi *et al.* (2019 ▸) the actual film thickness may be much smaller, the protein concentration much larger and the fraction of solvent bound in the first two hydration layers of the protein larger. Consequently, the concentrations of protein and salt present in the samples of Shi and coworkers may have been sufficient to allow vitrification at 163 K. More generally, where biomolecules preferentially accumulate at the air–solvent interface, and where the sample film is thinned by evaporation or blotting to contain only one or two layers of biomolecules, substantial reductions in the critical cooling rate may be expected and sample vitrification at elevated temperatures may be feasible.

## 5. Related literature   

The following references are cited in the supporting information for this article: Childs *et al.* (1973 ▸), Corruccini & Gniewek (1960 ▸), Ehrlich *et al.* (2015 ▸), Feistel & Wagner (2006 ▸) and Loerting *et al.* (2011 ▸).

## Supplementary Material

Summary of data from the literature and online sources used in making the estimates in this manuscript, and additional discussion of some points. DOI: 10.1107/S2052252520002560/ua5002sup1.pdf

## Figures and Tables

**Figure 2:**
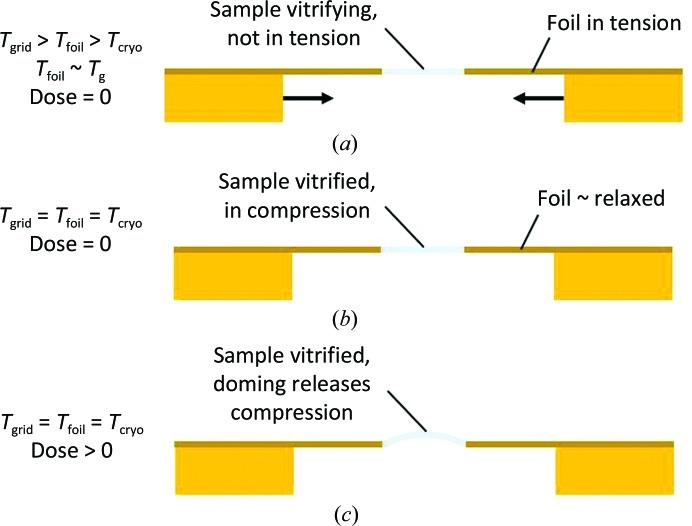
Schematic illustration of how compressive stress and doming may be generated during plunge-cooling of cryo-EM samples. (*a*) The sample and foil have cooled to *T*_g_ and the foil is under tensile stress between the warmer grid bars. (*b*) The sample, foil and grid have reached the final temperature *T*_cryo_; tensile stress in the foil associated with the transient temperature difference between the grid and foil has been released, and the sample is now under compression. (*c*) After receiving an electron dose *D*, radiation-induced creep in the presence of compressive stress has produced doming of the sample within the holes in the foil.

## Footnotes

1 This ignores the short timescale (∼1 ms) of cooling and any viscoelastic response of the sample on this timescale above *T*_g_.

## References

[bb1] Amann-Winkel, K., Böhmer, R., Fujara, F., Gainaru, C., Geil, B. & Loerting, T. (2016). *Rev. Mod. Phys.* **88**, 011002.

[bb2] Angell, C. A. (2002). *Chem. Rev.* **102**, 2627–2650. 10.1021/cr000689q 12175262

[bb3] Brilot, A. F., Chen, J. Z., Cheng, A., Pan, J., Harrison, S. C., Potter, C. S., Carragher, B., Henderson, R. & Grigorieff, N. (2012). *J. Struct. Biol.* **177**, 630–637. 10.1016/j.jsb.2012.02.003 PMC3322646 22366277

[bb4] Bullough, R. & Wood, M. H. (1980). *J. Nucl. Mater.* **90**, 1–21.

[bb5] Campbell, M. G., Cheng, A., Brilot, A. F., Moeller, A., Lyumkis, D., Veesler, D., Pan, J., Harrison, S. C., Potter, C. S., Carragher, B. & Grigorieff, N. (2012). *Structure*, **20**, 1823–1828. 10.1016/j.str.2012.08.026 PMC3510009 23022349

[bb6] Cheng, Y., Grigorieff, N., Penczek, P. A. & Walz, T. (2015). *Cell*, **161**, 438–449. 10.1016/j.cell.2015.03.050 PMC4409659 25910204

[bb40] Childs, G. E., Ericks, L. J. & Powell, R. L. (1973). *Thermal Conductivity of Solids at Room Temperature and Below*. Washington: National Bureau of Standards.

[bb41] Corruccini, R. & Gniewek, J. (1960). *Specific Heats and Enthalpies of Technical Solids at Low Temperatures*. Washington: National Bureau of Standards.

[bb7] Corruccini, R. & Gniewek, J. (1961). *Thermal Expansion of Technical Solids at Low Temperatures*. Washington: National Bureau of Standards

[bb42] Ehrlich, L. E., Feig, J. S. G., Schiffres, S. N., Malen, J. A. & Rabin, Y. (2015). *PLoS One*, **10**, e0125862. 10.1371/journal.pone.0125862 PMC4436132 25985058

[bb8] Faruqi, A. R. & McMullan, G. (2018). *Nucl. Instrum. Methods Phys. Res. A*, **878**, 180–190.

[bb43] Feistel, R. & Wagner, W. (2006). *J. Phys. Chem. Ref. Data*, **35**, 1021–1047.

[bb9] Glaeser, R. M. (2016). *Methods Enzymol.* **579**, 19–50. 10.1016/bs.mie.2016.04.010 27572722

[bb10] Glaeser, R. M., McMullan, G., Faruqi, A. R. & Henderson, R. (2011). *Ultramicroscopy*, **111**, 90–100. 10.1016/j.ultramic.2010.10.010 PMC3741612 21185452

[bb11] Hallbrucker, A. & Mayer, E. (1987). *J. Phys. Chem.* **91**, 503–505.

[bb12] Henderson, R. (2018). *Angew. Chem. Int. Ed.* **57**, 10804–10825.

[bb13] Jenniskens, P. & Blake, D. F. (1996). *Astrophys. J.* **473**, 1104–1113. 10.1086/178220 11539415

[bb14] Kriminski, S., Caylor, C. L., Nonato, M. C., Finkelstein, K. D. & Thorne, R. E. (2002). *Acta Cryst.* D**58**, 459–471. 10.1107/s0907444902000112 11856832

[bb15] Li, X., Mooney, P., Zheng, S., Booth, C. R., Braunfeld, M. B., Gubbens, S., Agard, D. A. & Cheng, Y. (2013). *Nat. Methods*, **10**, 584–590. 10.1038/nmeth.2472 PMC3684049 23644547

[bb45] Loerting, T., Bauer, M., Kohl, I., Watschinger, K., Winkel, K. & Mayer, E. (2011). *J. Phys. Chem. B*, **115**, 14167–14175. 10.1021/jp204752w 21879742

[bb16] Mayer, E. & Hallbrucker, A. (1987). *Nature*, **325**, 601–602.

[bb17] Moreau, D. W., Atakisi, H. & Thorne, R. E. (2019). *IUCrJ*, **6**, 346–356. 10.1107/S2052252519001878 PMC6503922 31098016

[bb18] Naydenova, K., Peet, M. J. & Russo, C. J. (2019). *Proc. Natl Acad. Sci. USA*, **116**, 11718–11724. 10.1073/pnas.1904766116 PMC6575631 31127045

[bb19] Ravelli, R. B. G., Theveneau, P., McSweeney, S. & Caffrey, M. (2002). *J. Synchrotron Rad.* **9**, 355–360. 10.1107/s0909049502014541 12409622

[bb20] Ripstein, Z. A. & Rubinstein, J. L. (2016). *Methods Enzymol.* **579**, 103–124. 10.1016/bs.mie.2016.04.009 27572725

[bb21] Röttger, K., Endriss, A., Ihringer, J., Doyle, S. & Kuhs, W. F. (1994). *Acta Cryst.* B**50**, 644–648. 10.1107/S0108768111046908 22267563

[bb22] Rubinstein, J. L. & Brubaker, M. A. (2015). *J. Struct. Biol.* **192**, 188–195. 10.1016/j.jsb.2015.08.007 26296328

[bb23] Russo, C. J. & Passmore, L. A. (2016*a*). *Curr. Opin. Struct. Biol.* **37**, 81–89. 10.1016/j.sbi.2015.12.007 PMC4863039 26774849

[bb24] Russo, C. J. & Passmore, L. A. (2016*b*). *J. Struct. Biol.* **193**, 33–44. 10.1016/j.jsb.2015.11.006 PMC4711342 26592474

[bb25] Shi, H., Ling, W., Zhu, D. & Zhang, X. (2019). *bioRxiv*, 824698.

[bb26] Shibata, T. (2013). *Handbook of Advanced Ceramics*, 2nd ed., edited by S. Somiya, pp. 113–123. Waltham: Academic Press.

[bb27] Tyree, T. J., Dan, R. & Thorne, R. E. (2018). *Acta Cryst.* D**74**, 471–479. 10.1107/S2059798318003078 PMC5930352 29717718

[bb28] Vinothkumar, K. R. & Henderson, R. (2016). *Q. Rev. Biophys.* **49**, e13. 10.1017/S0033583516000068 27658821

[bb29] Warkentin, M. A., Sethna, J. P. & Thorne, R. E. (2013). *Phys. Rev. Lett.* **110**, 015703. 10.1103/PhysRevLett.110.015703 23383808

[bb30] Wright, E. R., Iancu, C. V., Tivol, W. F. & Jensen, G. J. (2006). *J. Struct. Biol.* **153**, 241–252. 10.1016/j.jsb.2005.12.003 16434212

[bb31] Zheng, S. Q., Palovcak, E., Armache, J.-P., Verba, K. A., Cheng, Y. & Agard, D. A. (2017). *Nat. Methods*, **14**, 331–332. 10.1038/nmeth.4193 PMC5494038 28250466

