# Supplementary material for: Hypothesis for a mechanism of beam-induced motion in cryo-electron microscopy
Source: IUCrJ. 2020 Mar 26;7(Pt 3):416–21. doi: 10.1107/S2052252520002560 (PMC7201280; doi:10.1107/S2052252520002560)
Supplement: Supplementary file 1 [file m-07-00416-sup1.pdf]

IUCrJ

Volume 7 (2020)

Supporting information for article:

Hypothesis for a mechanism of beam-induced motion in cryo-electron microscopy

Robert E. Thorne

Here I summarize data from the literature and online sources used in making the estimates in this manuscript, and provide additional discussion of some points, including those raised by the referees.

S1. Can a foil slip relative to the supporting grid during cooling?

Differential contraction during cooling routinely causes delamination of thin film multilayers deposited by thermal or e-beam evaporation, indicating relative local motions at the interface between layers, so in general the answer should be yes. Whether this occurs in practice will depend on how the local tensile forces exerted by the foil compare with the local forces that hold the foil in place on the grid bars. The foil's tensile force will depend on the foil's average thickness and on its thermal contraction relative to the grid. The forces that hold the foil to the grid include dispersion and electrostatic forces and friction forces due to surface roughness. These forces can be reduced through appropriate surface preparation of the grid and foil, or by using intrinsically layered materials. In typical practice, foils will be fixed to the grids by frozen solvent, either from the sample or due to moisture adsorption from the ambient atmosphere. This could in principle be addressed with appropriate foil design, appropriate sample deposition and blotting procedures, and grid and backside foil coatings.

A simple test for foil slippage relative to the supporting grid is to repeatedly plunge and warm a dry grid + foil in a dry atmosphere. If the foil's thermal contraction between room temperature and cryogenic temperature is equal to or greater than that of the grid, and if foil buckling is observed at room temperature after several plunges, then the foil likely slipped relative to the grid.

S2. Thermal expansion coefficient of hexagonal ice I_h

Hexagonal ice I_h and low-density amorphous ice I_{LDA} have similar T=77 K densities (0.93 vs 0.937 g/cm^3 (Loerting *et al.*, 2011)), reflecting similar average coordinations of water molecules, and should have similar thermal expansion behavior over the temperature range where I_{LDA} remains metastable. Fig. S1, based on the data of Röttger *et al.* (Röttger *et al.*, 1994), shows values of cell parameters *a* and *c* for hexagonal ice, normalized by their values at T=10 K. *a* and *c* undergo identical fractional expansions, within experimental error. Between the glass transition temperature of pure water T_g~136 K and 90 K, the average linear expansion coefficient obtained from this data (with a 10 K reference temperature) is 1.5×10^{-5} K^{-1}, and the total fractional contraction is ~0.068%.

S3. Thermal expansion coefficients of metals used in cryoEM grids

Figure S2 shows data for the linear thermal expansion of metals commonly used in cryo-EM grids and foils versus temperature (Corruccini & Gniewek, 1961), in the temperature range of relevance for matching to the expansion of amorphous ice. The average coefficients of linear expansion between T_g~136 K and 90 K (with the 10 K reference temperature) are 1.39, 1.16, 1.18, 0.32, 0.70, 0.52, and 0.29 × 10^{-5} K^{-1} for Al, Cu, Au, Mo, Ni, Ti, and W, respectively. Al, Cu, and Au provide the closest match to the expansion of ice over this temperature range. Note that since overall expansions between 10 K and room temperature are less than 1%, these average coefficients of linear expansion are not appreciably affected by the choice of reference temperature.

S4. Heat capacities of ice and cryoEM grid materials

Figure S3 shows the specific heats of metals commonly used in cryoEM grids and foils versus temperature (Corruccini & Gniewek, 1960). Between 300 K and 90 K, the average specific heats are 0.744, 0.338, 0.121, 0.208, 0.359, 0.438, and 0.119 kJ / kg K^{-1} for Al, Cu, Au, Mo, Ni, T, and W, respectively.

Figure S4 shows the specific heat of water and of hexagonal ice versus temperature (Feistel & Wagner, 2006). The average specific heat between 273 K and 90 K is 1.47 kJ / kg K, and between 273 K and 300 K is 4.18 kJ/kg K.

Even though the specific heat per unit mass of water is much larger than that of the metals in Fig. S2, water has a much lower density, so on a per unit volume basis the specific heats are comparable. The average specific heats between 300 K and 90 K in J / cm^3 K are 2.01, 3.03, 2.34, 2.14, 3.20, 1.97, and 2.30 for Al, Cu, Au, Mo, Ni, T, and W, respectively; for hexagonal ice between 273 K and 90 K, the value is ~1.58.

The total amount of heat that must be removed from the metals to cool from 300 K to 90 K is just the average specific heat over that temperature range times the temperature difference. For water, the latent heat of fusion must be added to the total. The resulting total heat capacities per unit mass between 300 K and 90 K are 71.1, 25.4, and 715 kJ/kg for Cu, Au, and water. On a per unit volume basis (using the room temperature densities), these are 637, 491, and 715 J/cm^3.

S5. Thermal conductivities of ice and cryoEM grid materials

The thermal conductivities of Cu and Au are 385 and 314 W m^{-1} K^{-1} at 300 K, and on cooling to 90 K these increase by less than a factor of 1.5 (Childs *et al.*, 1973). The thermal conductivity of amorphous carbon is ~1 between 200 K and 100 K. The thermal conductivity of hexagonal ice increases linearly with decreasing temperature from 2.2 at 273 K, 3.5 at 177 K, and ~6 at 90 K (Ehrlich *et al.*, 2015).

References cited

Childs, G. E., Ericks, L. J. & Powell, R. L. (1973). *NBS Monogr.* **131**, 1–594.

Corruccini, R. & Gniewek, J. (1960). *NBS Monogr.* **21**, 1–20.

Corruccini, R. & Gniewek, J. (1961). Thermal expansion of technical solids at low temperatures.

Ehrlich, L. E., Feig, J. S. G., Schiffres, S. N., Malen, J. A. & Rabin, Y. (2015). *PLoS One*. **10**, 1–19.

Feistel, R. & Wagner, W. (2006). *J. Phys. Chem. Ref. Data*. **35**, 1021–1047.

Loerting, T., Bauer, M., Kohl, I., Watschinger, K., Winkel, K. & Mayer, E. (2011). *J. Phys. Chem. B*. **115**, 14167–14175.

Röttger, K., Endriss, A., Ihringer, J., Doyle, S. & Kuhs, W. F. (1994). *Acta Cryst. B*. **50**, 644–648.

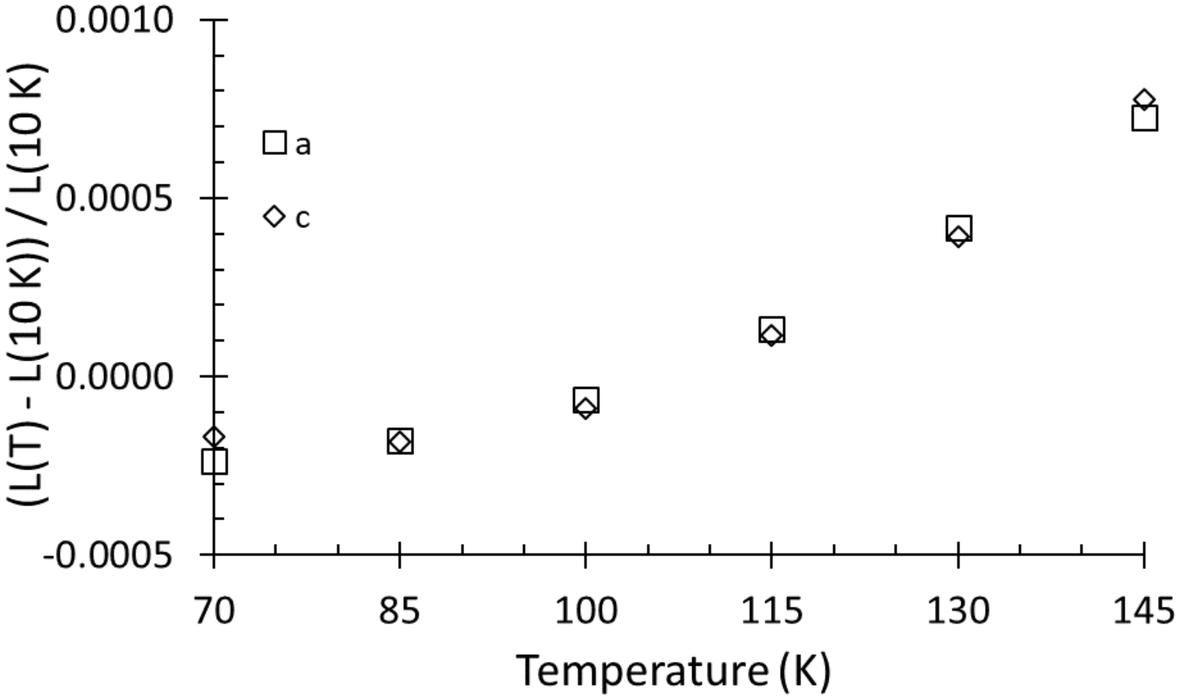

Figure S1 Unit cell parameters a and c, normalized by their values at T=10 K, for hexagonal ice I_h. Below 70 K, the cell parameters are roughly constant.

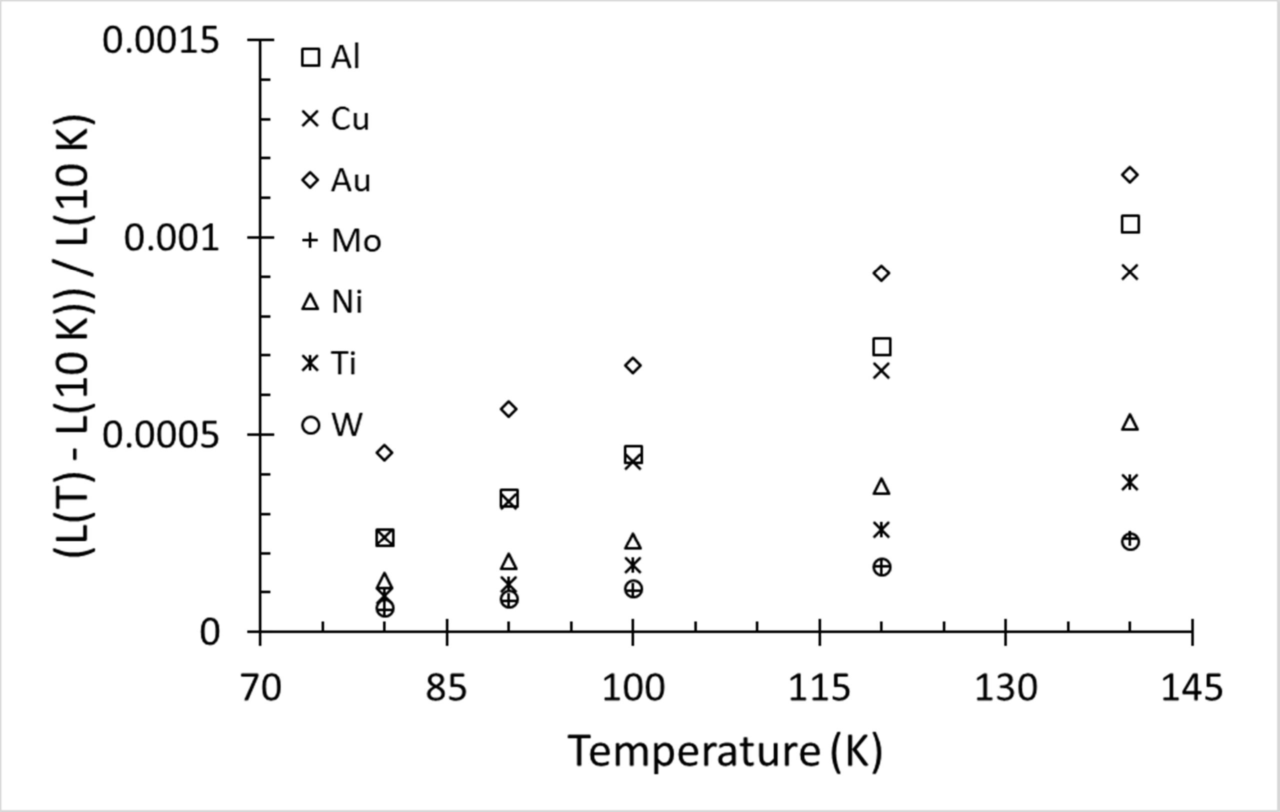

Figure S2 Linear thermal expansion relative to T=10 K for several metals used in cryo- EM supports. Below 70 K, these metals continue contracting.

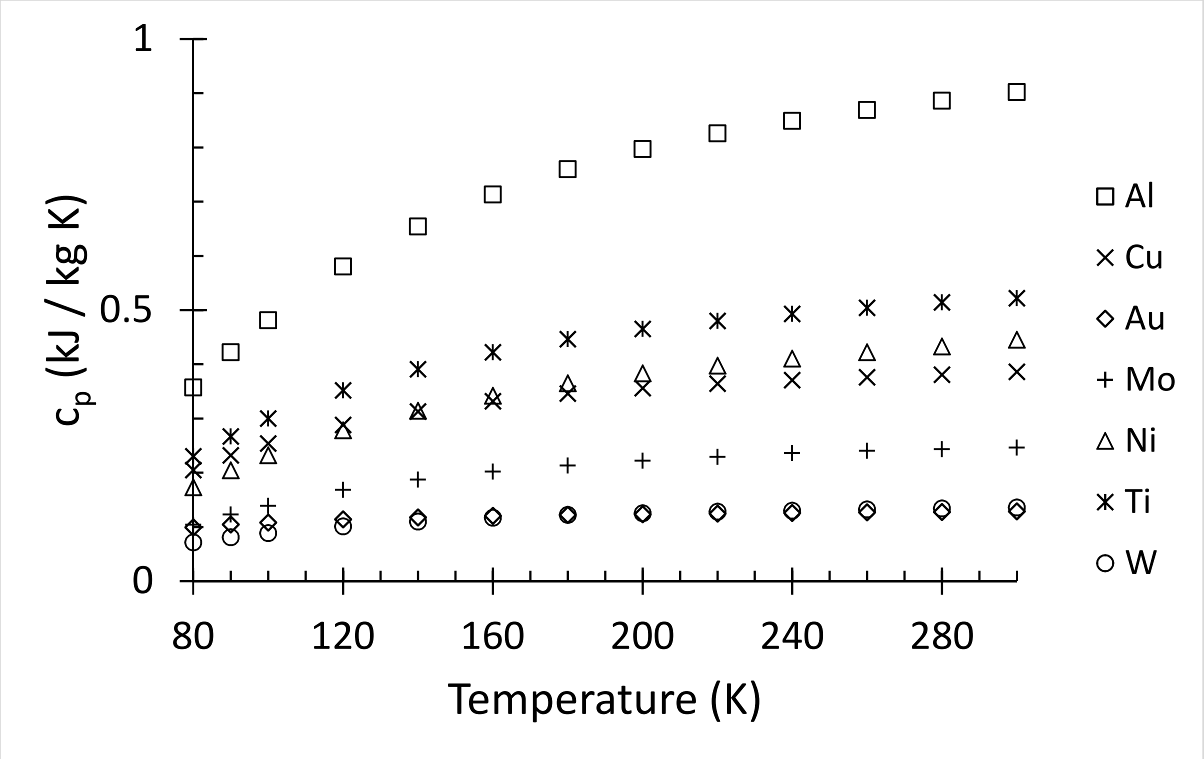

Figure S3 Specific heats of common metals used in cryo-EM supports versus temperature.

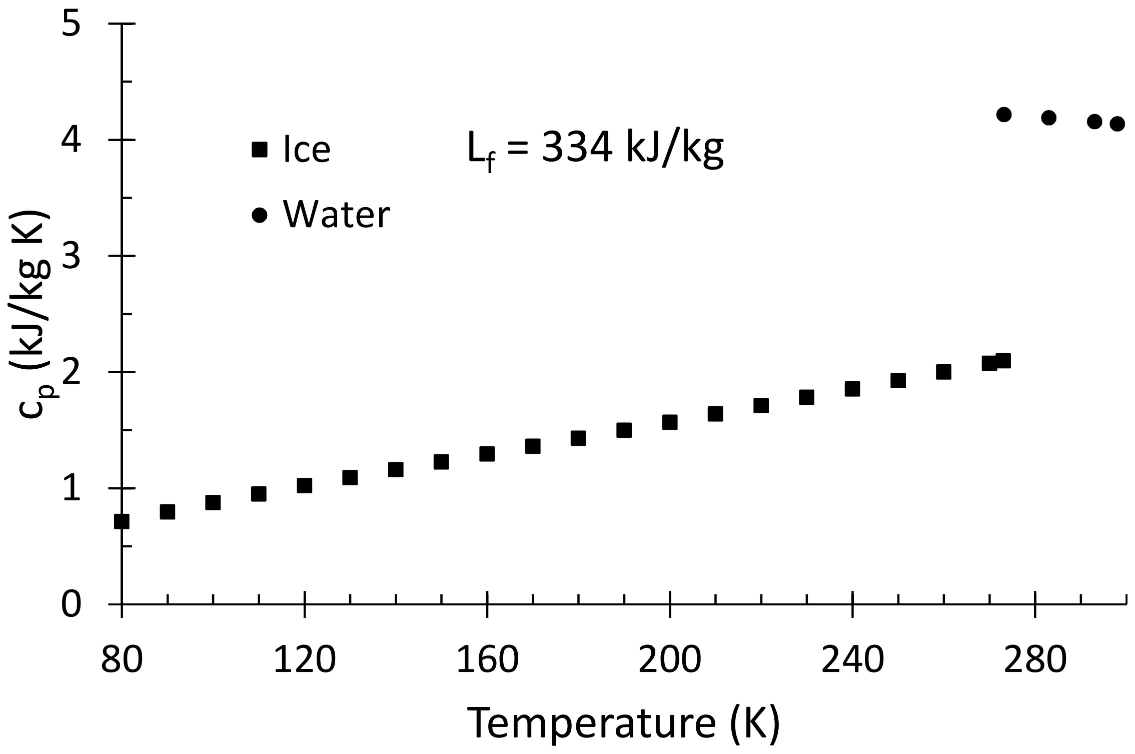

Figure S4 Specific heats of water (for T>273.15 K) and hexagonal ice.
